# Fetal monitoring using a wearable ultrasound patch for high-risk pregnancies

**DOI:** 10.21203/rs.3.rs-7715480/v1

**Published:** 2025-10-09

**Authors:** Geonho Park, Yizhou Bian, Hao Huang, Sai Zhou, Siyu Qin, Muyang Lin, Xinyi Yang, Aaron Lee, Anand Ramkumar, Mariana Tome, Jayne Lander, Xiangjun Chen, Shenghan Wang, Pranavi Bheemreddy, Liam Stanton, Mabel Shehada, Ruotao Wang, Alexa Roa, Chengchangfeng Lu, Wentong Yue, Ray S. Wu, Xiaoxiang Gao, Hongjie Hu, Amer Yaghi, Mark Liu, Lawrence Impey, Sally L. Collins, Aris T Papageorghiou, Louise C. Laurent, Antoniya Georgieva, Sheng Xu

**Affiliations:** 1 Aiiso Yufeng Li Family Department of Chemical and Nano Engineering, University of California San Diego, La Jolla, CA 92093, USA.; 2 Materials Science and Engineering Program, University of California San Diego, La Jolla, CA 92093, USA.; 3 Department of Electrical and Computer Engineering, University of California San Diego, La Jolla, CA 92093, USA.; 4 Shu Chien–Gene Lay Department of Bioengineering, University of California San Diego, La Jolla, CA 92093, USA.; 5 Nuffield Department of Women’s and Reproductive Health, University of Oxford, Oxford OX3 9DU, UK.; 6 Qualcomm Institute Makerspace, University of California San Diego, La Jolla, CA 92093, USA.; 7 Department of Obstetrics, Gynecology, and Reproductive Sciences, University of California San Diego, La Jolla, CA 92093, USA.; 8 Department of Radiology, University of California San Diego, La Jolla, CA 92093, USA.; 9 These authors contributed equally to this work: Geonho Park, Yizhou Bian, Hao Huang, Sai Zhou.

## Abstract

The rapid and complex nature of fetal development requires meticulous prenatal monitoring to ensure optimal pregnancy outcomes^1^. Cardiotocography, which continuously records the fetal heart rate and uterine contractions, often leads to inaccurate diagnoses and unnecessary interventions^2^. Ultrasonography is a cornerstone of fetal monitoring and diagnosis, but it is highly dependent on specialized sonographers, limiting its availability^3^. Additionally, current ultrasound methods provide only snapshot evaluations^4^. Even in very high-risk pregnancies, it is rare to have fetal ultrasound assessments more than once per day^5^. Here we report a wearable ultrasound patch (UPatch) for continuous and autonomous fetal monitoring. The UPatch can acquire anatomical structures and blood flow velocities, with signal qualities comparable to those of handheld clinical ultrasound devices. Real-time image segmentation allows the autonomous tracking of target vessels and thus the acquisition of continuous blood flow spectra during fetal and maternal movements without a sonographer. We validated the UPatch accuracy on 62 pregnancies, and the continuous monitoring data on 52 pregnant women aligned with stratified perinatal conditions, including healthy, small for gestational age, large for gestational age, gestational diabetes, pre-eclampsia, and gestational hypertension. The UPatch introduces new capabilities for prenatal care and offers critical evidence for studying fetal complications in high-risk pregnancies.

The physiological maturation of the fetus in the uterus is supported by cardiovascular exchanges between the maternal, placental, and fetal circulations^6^. Any disruption of this delicate exchange system can precipitate fetal complications, such as hypoxia^7^, intrauterine growth restriction^8^, and cerebral palsy^9^, with stillbirth^10^ being the most extreme outcome. Despite decades of research, many such fetal complications remain undiagnosed and unexplained, with existing methods relying mostly on postmortem investigations^11,12^. Continuous monitoring of fetal anatomy and hemodynamics throughout pregnancy not only provides insights into the pathophysiological mechanisms of fetal complications but also allows the early detection and mitigation of emerging issues, helping to prevent adverse outcomes (Supplementary Discussion 1)^1,13^.

Continuous fetal monitoring currently relies predominantly on cardiotocography, which measures the fetal heart rate and uterine contractions (Supplementary Fig. 1 and Supplementary Discussions 2 and 3)^14,15^. However, given the complexity of fetal physiology, these measurements do not correlate well with fetal health and yield false positive rates up to 50% (Supplementary Discussion 3)^2,16–18^. Other continuous monitoring devices based on electrocardiography^19^, pulse oximetry^20^, or accelerometry^21^ cannot reliably differentiate fetal from maternal signals and are not commonly used in clinical practice (Supplementary Fig. 1 and Supplementary Discussion 2). Ultrasonography is widely used to measure fetal biometry and blood flow (Supplementary Discussion 4)^22–24^, and standardized reference indices have been established throughout gestation to evaluate fetal health (Supplementary Discussion 5)^25–28^. However, conventional ultrasonography is heavily dependent on the skills of the operator, and measurements are limited to snapshots that can fail to capture the patterns and critical moments of fetal hemodynamics^29,30^. Although wearable ultrasound devices have been developed for the continuous monitoring of physiological signals^31–34^, current prototypes are unsuitable for fetal monitoring due to weak blood flow signals from deep fetal vessels and signal loss during large fetal movements.

Here we report a wearable ultrasound patch (UPatch) that can continuously image the fetus and autonomously measure fetal blood flow in real time (Supplementary Discussion 6). To enhance the signal-to-noise ratio and imaging resolution, we integrated transducers fabricated by super multipass dicing, as well as an acoustic lens and a soft Faraday cage. To identify and track moving targets, we used segmentation-based algorithms to place sample gates in vessel images. Blood flow spectra can be acquired continuously and autonomously in real time, even during large fetal movements. We conducted a prospective study with 62 pregnant participants to validate the accuracy of the UPatch against a handheld clinical ultrasound device. The mean differences ± standard deviations between these two devices were −0.019 ± 0.274 for the systolic-to-diastolic ratio, −0.010 ± 0.117 for the pulsatility index, −0.004 ± 0.044 for the resistance index, and 0.95 ± 2.91 beats per minute (bpm) for the fetal heart rate, demonstrating excellent agreement between the devices. Furthermore, we used the UPatch to continuously monitor the umbilical cord blood flow in 52 participants, generating longitudinal measurements that distinguished transient fluctuations from sustained compromise. Doppler indices exhibited monotonic relationship with gestational age and differentiated high-risk from healthy pregnancies. In a pre-eclamptic case, the UPatch revealed the severity of intrauterine growth restriction, leading to Cesarian delivery to prevent stillbirth.

## Design of the UPatch

**Fig. 1 |:**
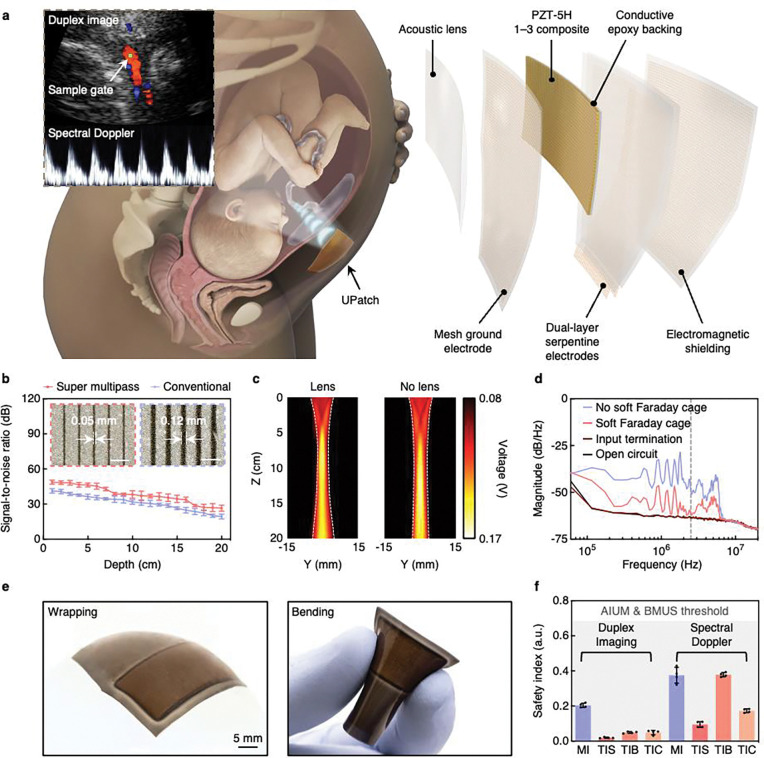
Overview of the UPatch. **a**, Schematics of the working principle for fetal monitoring (left) and a structurally exploded view (right). The UPatch is attached to the maternal abdomen to monitor fetal anatomical structures and hemodynamics continuously with duplex imaging. A sample gate, indicated by the green dot, provides blood flow measurements from fetal vessels. A diverging beam is used for imaging, and a focused beam for blood flow measurement. The UPatch is multilayered to enhance its signals from red blood cells and minimize its form factors. **b**, Comparison of signal-to-noise ratios between the conventional method and transducers diced with the super multipass method (n = 12). The insets are optical images showing the results from the two methods. Scale bars = 0.5 mm. **c**, Acoustic fields measured using a hydrophone in the elevational plane of the UPatch with (left) and without (right) an acoustic lens. **d**, The power spectral density analysis of the UPatch with and without the soft Faraday cage, input termination at 50 Ω, and open circuit of the backend system. The soft Faraday cage reduces noise coupling from electromagnetic interference by 11.7 dB/Hz at 2.5 MHz (gray dashed line), the center frequency of the UPatch. **e**, Photographs of the UPatch demonstrating its mechanical compliance in wrapping (left) and bending (right) shapes. **f**, UPatch safety measurements. The mechanical index (MI), soft tissue thermal index (TIS), bone thermal index (TIB), and cranium thermal index (TIC) were all well below the thresholds of 0.7 recommended by the AIUM^44^ and BMUS^45^ for continuous monitoring (n = 4). Error bars in **b** and **f** show ± standard deviations.

Fetal structures and vessels vary in size and are distributed over a wide range of depths in the uterus (Fig. 1a, left)^35^. Brightness-mode imaging is based on echoes from tissue interfaces and distributed scattering within tissues^36^. On the other hand, color Doppler imaging and spectral Doppler waveforms of blood flow are based on the much weaker echoes from red blood cells^36,37^.

We adopted three strategies to strengthen the echoes from red blood cells in deep fetal vessels. First, the UPatch contains a 2.5-MHz PZT-5H 1–3 composite with a conductive epoxy backing layer at a pitch of 0.65 ultrasound wavelength (Fig. 1a, right). We used super multipass dicing, which reduced the kerf and thus increased the element width by 58% (Fig. 1b, Methods, Supplementary Fig. 2, and Supplementary Discussion 7). This improved the signal-to-noise ratio by 7.2 dB without increasing the crosstalk between adjacent elements (Fig. 1b and Supplementary Figs 3 and 4). Second, we designed an acoustic lens to improve elevational focusing, which reduced the focal beamwidth by 25% and thus enhanced the spatial resolution and color Doppler signal sensitivity (Fig. 1c, Methods, Supplementary Figs 5–8, and Supplementary Discussion 8)^38^. Additionally, the acoustic lens moved the beam elevational focal depth from 14 to 10 cm, on par with clinical devices, to balance the spatial resolution throughout the entire uterus (Fig. 1c and Supplementary Fig. 6). Third, we developed a soft Faraday cage by connecting the electromagnetic shielding layer to the mesh ground electrode layer using vertical interconnect accesses (Methods, Supplementary Figs 9 and 10, and Supplementary Discussion 9). The cage fully encapsulated the elements and reduced the electromagnetic interference by 11.7 dB/Hz (Fig. 1d and Supplementary Figs 11–13)^39^. Additionally, we used two signal electrode layers that can individually address each element with a minimal electrode footprint (Supplementary Fig. 9). The UPatch was mechanically compliant (Fig. 1e) and showed negligible constraints on the movement of pregnant participants in various positions (Supplementary Fig. 14).

To quantify the UPatch performance, we characterized its brightness, color Doppler, and spectral Doppler modes following the Institute of Physics and Engineering in Medicine protocols (Methods)^40^. On a phantom filled with monofilament wires and tissue-mimicking contrast targets, the axial and lateral resolutions, dynamic range, and contrast-to-noise ratio of the UPatch in brightness mode were comparable to those of a handheld clinical ultrasound device (Supplementary Fig. 15)^41^. On a flow phantom with blood-mimicking fluids, the color Doppler sensitivity and the spectral Doppler accuracy of the UPatch at different depths and velocities were similar to those of the clinical device (Supplementary Figs 16 and 17)^42^. On a customized umbilical cord flow phantom, the UPatch reliably acquired flow signals regardless of phantom orientation (Supplementary Figs 18–20 and Supplementary Discussion 10). On a healthy adult participant, all three modes of the UPatch and the clinical device were comparable in quality (Supplementary Figs 21 and 22).

## Acoustic safety characterizations

Minimal acoustic intensity is necessary for the fetus to avoid potential side effects associated with ultrasound exposure^43^. We examined the biological effects of the UPatch in vitro in all three modes with strict adherence to the guidelines from the American Institute of Ultrasound in Medicine (AIUM)^44^, British Medical Ultrasound Society (BMUS)^45^, and Food and Drug Administration (FDA)^46^ (Methods and Supplementary Discussion 11). The maximum spatial average temporal average intensity was 0.72 mW/cm^2^ (< 20 mW/cm^2^ is recommended by the FDA^46^, and 5 mW/cm^2^ is the value reported for the GE Corometrics 250CX^47^). Furthermore, the derated spatial peak pulse average intensity was 16.42 W/cm^2^ (< 190 W/cm^2^ is recommended by the FDA^46^), the derated spatial peak temporal average intensity was 58.5 mW/cm^2^ (< 94 mW/cm^2^ is recommended by the FDA^46^), and the mechanical index was 0.38 (< 1.9 is recommended by the FDA^46^ and ≤ 0.7 is recommended by the BMUS^45^) (Fig. 1f and Supplementary Discussion 12).

We also evaluated the thermal characteristics of the UPatch^48,49^. Surface temperatures were tested using a thermocouple on a phantom and in air (Methods). In both experiments, the temperature increase was < 0.7 °C during 48 h of continuous UPatch activation (Supplementary Fig. 23 and Supplementary Discussion 12). To calculate the thermal index, acoustic power was measured and cross-checked using a hydrophone and radiation force balance (Methods, Supplementary Figs 24 and 25, and Supplementary Discussion 11)^49^. The soft tissue thermal index, bone thermal index, and cranium thermal index were 0.02, 0.05, and 0.05 for duplex (combined brightness and color Doppler) imaging, and 0.1, 0.38, and 0.17 for spectral Doppler, respectively (Fig. 1f). All results were well below the maximum level of 0.7 recommended by the AIUM and BMUS for continuous monitoring (Supplementary Discussion 12)^44,45^.

## Fetal monitoring and validation

**Fig. 2 |:**
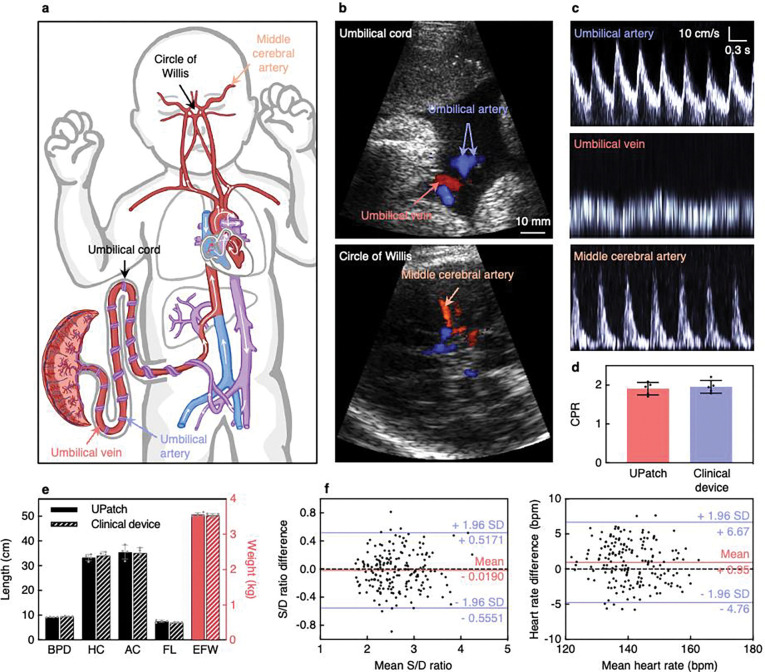
Measurements and validations of the UPatch for fetal monitoring. **a**, Schematics illustrating the fetal cardiovascular system, with labels indicating key vessels for pregnancy monitoring. **b**, Duplex images from the umbilical cord and circle of Willis recorded with the UPatch. The images share the same scale bar. **c**, Blood flow spectra from three key vessels recorded with the UPatch. The spectra share the same scale bar. **d**, Comparison of the cerebroplacental ratio (CPR) measured with the UPatch and a handheld clinical ultrasound device (n = 5). **e**, Comparison of fetal biometry measured with the UPatch and a handheld clinical ultrasound device. Biparietal diameter (BPD), head circumference (HC), abdominal circumference (AC), and femur length (FL) were used to calculate estimated fetal weight (EFW) (n = 4)^51^. **f**, Bland–Altman plots for the systolic-to-diastolic ratio (S/D ratio, left) and fetal heart rate (right) measured using the UPatch and a handheld clinical ultrasound device (Voluson E10, GE). Solid red lines are the mean differences between the two devices, solid blue lines are 95% limits of agreement (1.96 standard deviations above and below the mean differences), and black dashed lines are the zero difference between the two devices. Each plot has three measurement pairs by repeating three times with the same devices on the same participant for each of the 62 participants. Error bars in **d** and **e** show ± standard deviations.

Duplex imaging can reveal fetal anatomical abnormalities, quantify fetal biometry, and facilitate the recording of spectral Doppler waveforms^4^. Correcting the phase aberration induced by the maternal abdomen allowed the acquisition of high-quality signals from the UPatch (Supplementary Fig. 26 and Supplementary Discussion 13). The UPatch was able to image the two arteries and a vein in the helical umbilical cord and the middle cerebral artery arising from the circle of Willis (Fig. 2a,b). Detailed blood flow velocities were acquired from all of these vessels (Fig. 2c and Supplementary Discussion 5). The cerebroplacental ratio (the pulsatility index of the middle cerebral artery divided by that of the umbilical artery) quantifies the fetal brain-sparing effect during prolonged hypoxia or in placental insufficiency, and the measurements acquired by the UPatch and a clinical device were comparable (Fig. 2d and Supplementary Discussion 5)^50^. The UPatch accurately measured anatomical structures (such as the biparietal diameter, head circumference, abdominal circumference, and femur length), providing a fetal weight estimate for the diagnosis of growth-related complications (Fig. 2e, Methods, and Supplementary Fig. 27)^51^.

The UPatch accuracy was validated against a clinical device on 62 participants with various vessel depths, orientations, and placental locations (Table 1, Methods, and Supplementary Figs 28–30). From the spectral Doppler waveforms, an envelope extraction algorithm can derive peak systolic velocity, end diastolic velocity, and fetal heart rate to calculate the systolic-to-diastolic ratio, pulsatility index, and resistance index (Supplementary Fig. 31 and Supplementary Discussion 5)^52^. Based on Bland–Altman analysis, the mean differences ± standard deviations between the UPatch and clinical device were −0.019 ± 0.274 for the systolic-to-diastolic ratio, −0.010 ± 0.117 for the pulsatility index, −0.004 ± 0.044 for the resistance index, and 0.95 ± 2.91 bpm for the fetal heart rate (Fig. 2f and Supplementary Fig. 32).

## Autonomous vessel tracking

The umbilical cord facilitates gas exchange and nutrient transfer between the placenta and fetus^6^. Spectral Doppler waveforms of the umbilical blood flow can monitor the fetoplacental circulation (Supplementary Discussions 4 and 5)^8^. However, the umbilical cord is a moving target due to fetal and maternal movements, respiration, and uterine contractions (Supplementary Discussion 14)^24^. In clinical practice, sonographers manually adjust the color-flow box and set a sample gate to acquire spectral Doppler signals from the target vessel, which is time-consuming and operator-dependent (Supplementary Fig. 33)^53^. In low-resource settings where the incidence of pregnancy complications remains high, such sonographers are often limited in number^54,55^.

**Fig. 3 |:**
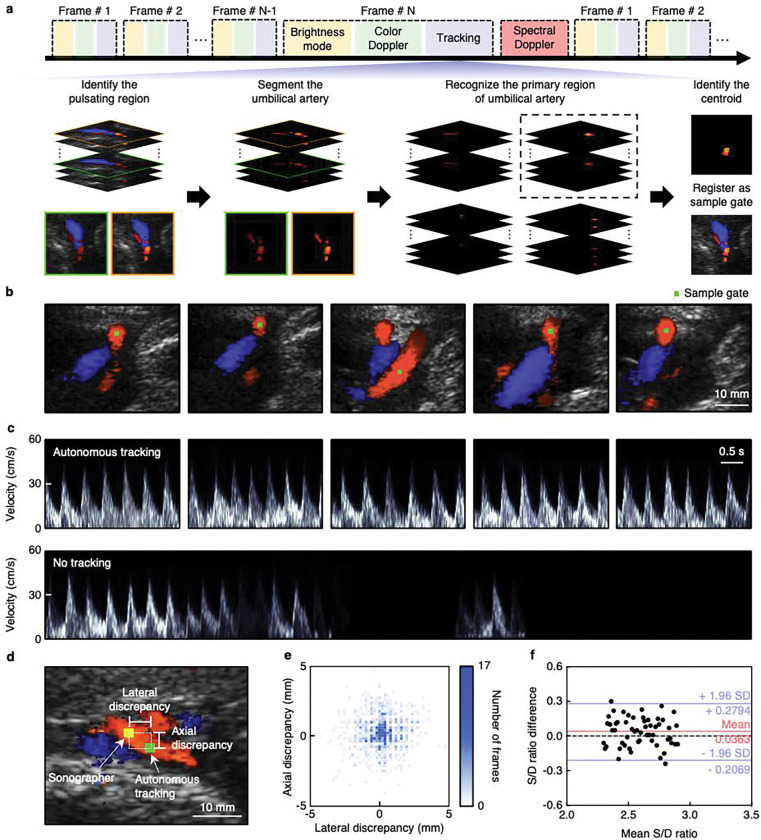
Autonomous vessel tracking by image segmentation. **a**, Beamforming sequence and working principle of the image segmentation-based vessel tracking algorithm. The tracking algorithm is integrated between the duplex imaging and spectral Doppler (top). The algorithm identifies the pulsating regions in consecutive frames, segments the umbilical artery, recognizes the primary region, and registers the spatial centroid as the sample gate (bottom). Images with green and orange boundaries represent the end diastole and peak systole, respectively. In these two images, the blue regions are similar, indicating the umbilical vein, whereas the red regions differ in intensity, indicating the pulsating umbilical artery. Among the four segmented areas, the primary region is defined as the largest segmented area (denoted with the black dashed box), which has the strongest signal intensity. **b**, Tracking a moving umbilical artery and registering a sample gate using the UPatch with the autonomous algorithm. All images share the same scale bar. **c**, The autonomous algorithm allows the measurement of blood flow from a moving vessel continuously (top). Without the autonomous tracking algorithm, a predefined sample gate results in signal loss due to vessel movements (bottom). The spectra share the same scale bar. **d**, A representative image showing the difference between the sample gate labeled by the autonomous tracking algorithm and by a sonographer. **e**, Summary of the lateral and axial discrepancies in the sample gates labeled by the autonomous tracking algorithm and by a sonographer. **f**, Bland–Altman plot for the systolic-to-diastolic ratios (S/D ratio) measured by the autonomous tracking algorithm and a sonographer. The solid red line is the mean difference between the two methods, the solid blue lines are 95% limits of agreement (1.96 standard deviations above and below the mean differences), and the black dashed line is the zero difference between the two methods.

To reduce the need for manual operators and the corresponding healthcare burden, we developed an image segmentation-based algorithm that can autonomously identify and track moving vessels (Fig. 3a and Supplementary Discussion 15). We used a diverging beam to cover a large field of view, ensuring that the moving vessel remains consistently visible within the ultrasonographic window (Supplementary Fig. 34)^56^. The UPatch stably acquired the umbilical cord signals without repositioning across common maternal postures (Supplementary Fig. 35). By segmenting the reconstructed duplex images, the tracking algorithm identifies pulsatile signals by calculating the variance in color Doppler pixel intensity over several consecutive frames (Fig. 3a, Supplementary Fig. 36, and Supplementary Discussions 15 and 16). Then, the algorithm identifies the primary region of the pulsatile signals and registers the spatial centroid of the primary region as the sample gate, where a focused beam is directed to acquire blood flow spectra with enhanced signal intensity (Fig. 3a and Supplementary Figs 37 and 38)^56^. This autonomous sample gate registration enabled continuous blood flow monitoring of a moving vessel in real time (Fig. 3b,c, Supplementary Video 1, and Supplementary Discussion 17). Conversely, without the tracking algorithm, blood flow signals were lost during vessel movements (Fig. 3c).

To validate the accuracy of the autonomous tracking algorithm, we compared its sample gates with those registered by a sonographer in a double-blind test (Fig. 3d, Methods, and Supplementary Discussion 18). We found that 91.9% and 90.5% of the sample gates were within a 2-mm discrepancy (an accepted clinical standard) in the lateral and axial directions, respectively (Fig. 3e and Supplementary Discussion 19)^4^. In a separate test, the sample gates registered by the tracking algorithm were considered accurate by three sonographers in 94.2% of the images acquired using the clinical device (with some images affected by fast cord movements), and in 91.2% of the images acquired using the UPatch (due to slight differences in image quality, Methods, Supplementary Fig. 39, and Supplementary Discussion 18). We used Bland–Altman analysis to compare the systolic-to-diastolic ratio from the sample gates acquired by the tracking algorithm and a sonographer (Methods and Supplementary Discussion 18). The mean difference ± standard deviation was only 0.036 ± 0.124 (Fig. 3f).

## Continuous monitoring of pregnant participants

The continuous monitoring of umbilical artery blood flow is important for assessing fetal growth restriction (Supplementary Discussions 4 and 5)^5^. Abnormal waveforms (such as absent or reversed end diastolic velocities) often indicate fetal pathology due to placental insufficiency, and even subtler abnormalities (such as increased Doppler indices) indicate an elevated risk^57,58^. Continuous monitoring of these changes can inform the optimal delivery time, helping avoid both premature birth, which may lead to postnatal complications^59^, and delayed birth, which heightens the risk of asphyxia, cerebral palsy, and stillbirth^60^.

**Fig. 4 |:**
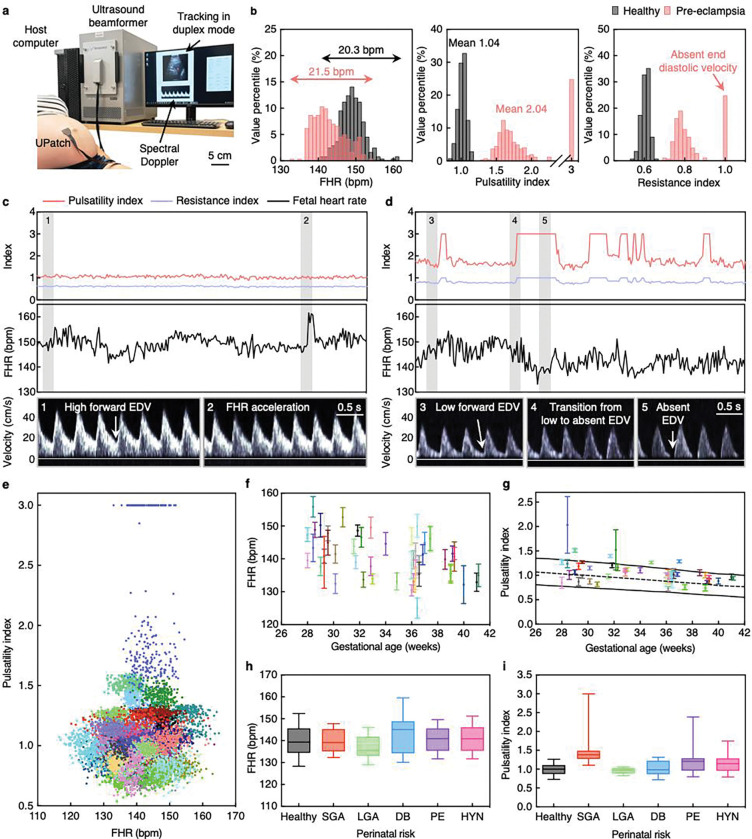
Continuous monitoring of pregnant participants. **a**, Photograph of the measurement setup, with participants in a semi-recumbent position. The UPatch was laminated on the maternal abdomen and connected to an ultrasound beamformer and a host computer, which displays the duplex image, tracks the sample gate, and measures spectral Doppler signals from the umbilical artery. **b**, Histograms of the fetal heart rate (FHR, left), pulsatility index (middle), and resistance index (right) from the healthy (grey) and pre-eclamptic (red) participants. **c**, Pulsatility index, resistance index (top), and FHR (middle) recording from the healthy participant. Spectral Doppler signals from the shaded regions (bottom). The spectra share the same scale bar. **d**, Pulsatility index, resistance index (top), and FHR (middle) recording from the pre-eclamptic participant. Spectral Doppler signals from the shaded regions (bottom). The spectra share the same scale bar. **e**, Scatterplot of pulsatility index against FHR with each color corresponding to an individual participant. **f**, FHR plotted against gestational age. **g**, Pulsatility index plotted against gestational age. The black dashed line is the 50^th^ percentile, and the solid black lines are the 5^th^ and 95^th^ percentiles of a widely used reference population^26^. The color schemes in **e**, **f**, and **g** are the same. Error bars in **f** and **g** show ± standard deviations. **h**,**i**, Box plots of (**h**) FHR and (**i**) pulsatility index stratified by perinatal condition: healthy (31 participants, 217 data points), small for gestational age (SGA; 7 participants, 49 data points), large for gestational age (LGA; 3 participants, 21 data points), diabetes (DB; 6 participants, 42 data points), preeclampsia (PE; 7 participants, 49 data points), and maternal hypertension (HYN; 10 participants, 70 data points). Each box represents the interquartile range (25^th^ to 75^th^ percentiles), the whiskers denote the 5^th^ to 95^th^ percentile range, and the midline indicates the median. Each participant’s data was segmented into 10-min intervals (n = 7 per participant).

We used the UPatch to compare a healthy (32 weeks 6 days) and a pre-eclamptic (28 weeks 3 days gestational age) participant. The participants were in a semi-recumbent position with the UPatch placed on the maternal abdomen to monitor the blood flow continuously at the placental cord insertion (Fig. 4a and Supplementary Discussion 20). We derived the fetal heart rate and Doppler indices, including resistance index, pulsatility index, and systolic-to-diastolic ratio, which are independent of the ultrasound incident angle (Supplementary Discussion 21)^22^. Both participants showed similar fetal heart rate patterns (Fig. 4b–d). The average fetal heart rates were 149.5 bpm for the healthy participant and 143.3 bpm for the pre-eclamptic participant, with variabilities of 20.3 and 21.5 bpm, respectively (Fig. 4b and Supplementary Discussion 22). The UPatch could detect transient fetal heart rate accelerations, which indicate a healthy fetal state (Fig. 4c, Supplementary Fig. 40, and Supplementary Discussion 3)^61^. However, the Doppler indices of the participants showed distinct features. There was minimal variability in the Doppler indices of the healthy participant, indicating stable blood flow (Fig. 4b,c and Supplementary Fig. 40). The mean resistance index of 0.62, pulsatility index of 1.04, and systolic-to-diastolic ratio of 2.61 aligned with the 50^th^ percentile of established reference norms, indicating a healthy fetus (Fig. 4b, Supplementary Fig. 40, and Supplementary Discussion 22)^26^. In contrast, the pre-eclamptic participant showed large fluctuations in the Doppler indices, and the end diastolic velocity was absent 24.9% of the time (Fig. 4b,d and Supplementary Fig. 41). The mean resistance index of 0.85, pulsatility index of 2.04, and systolic-to-diastolic ratio of 5.11 were above the 97.5^th^ percentile, suggesting severe placental dysfunction (Fig. 4b, Supplementary Fig. 41, and Supplementary Discussion 22)^26^. Following the detection of compromised fetal health using the UPatch, the pre-eclamptic participant underwent intensive monitoring, and the baby was delivered via Cesarean section 4 days later.

Current obstetric practice relies on a two-step process of cardiotocography for fetal heart rate and separate manual Doppler ultrasound for Doppler indices (Supplementary Discussion 23)^5^. We used the UPatch to continuously monitor both signals simultaneously in 52 pregnancies (Supplementary Figs 40–91 and Supplementary Discussion 24). Cohort analysis showed that the fetal heart rate and Doppler indices are not correlated, highlighting their potential to provide complementary clinical insights (Fig. 4e). Additionally, continuous monitoring results revealed that transient physiological fluctuations can temporarily alter Doppler indices to abnormal values (Supplementary Figs 40–95 and Supplementary Discussion 23). With conventional ultrasound devices, these transient fluctuations may be misinterpreted as pathological and trigger unnecessary interventions, whereas continuous monitoring with the UPatch establishes a personalized baseline and temporal context to distinguish transient fluctuations from sustained compromise. While fetal heart rate decelerations were observed, the substantial beat-to-beat variability and absence of a defined gestational reference trend make interpretation challenging (Fig. 4f)^62^. In contrast, Doppler indices exhibited monotonic relationships with gestational age, consistent with established reference ranges^26^, supporting their use as objective, clinically actionable information for assessing fetal vascular resistance and identifying high-risk pregnancies (Fig. 4g and Supplementary Fig. 96). The data were also stratified by perinatal conditions, including healthy, small for gestational age, large for gestational age, gestational diabetes, pre-eclampsia, and gestational hypertension (Fig. 4h,i). Fetal heart rate distributions overlapped substantially among all conditions, showing minimal discrimination (Fig. 4h). In contrast, Doppler indices differed markedly, particularly for small for gestational age and pre-eclampsia, illustrating the UPatch’s potential to identify high-risk pregnancies in real time (Fig. 4i and Supplementary Fig. 97).

## Discussion

The UPatch can visualize fetal anatomy and measure fetal heart rate and blood flow with high sensitivity and accuracy, offering complementary insights to existing approaches to evaluate fetal health. Whereas standard clinical regimens mandate periodic blood flow assessments performed by sonographers, typically at weekly intervals in outpatient settings and up to three times per week for inpatients, these evaluations are labor-intensive and prone to errors^57^. In contrast, the UPatch adheres directly to the maternal abdomen for hands-free use and employs an image segmentation algorithm to differentiate and track blood vessels autonomously, enabling continuous and accurate blood flow measurements without the need for a sonographer. The UPatch represents a transformative tool for improving outcomes in high-risk pregnancies and expanding access to quality prenatal care.

Several strategies could be pursued to improve the UPatch further. First, the tracking algorithm cannot generate uninterrupted spectral Doppler waveforms during motion because vessel tracking and spectral Doppler acquisition occur sequentially. This problem can be solved by integrating a separate image segmentation algorithm to detect signal loss in real time, allowing the system to pause the spectral Doppler sequence and track the target vessel in the duplex image. Leveraging advanced graphics processing units would substantially accelerate computation, enabling simultaneous duplex imaging and spectral Doppler acquisition (Supplementary Discussion 17)^63,64^.

Second, the UPatch currently needs to be wired to a backend system (Vantage 256, Verasonics) for both power and high-bandwidth data transmission, like many existing wearable devices^31,34,65–69^. While this configuration is appropriate for inpatient settings, where continuous monitoring is critical for assessing fetal health and optimizing delivery timing, it limits mobility and broader applicability. Future developments could incorporate wireless circuits to expand the range of clinical scenarios. A compact circuit was recently developed to interface a transducer array with a single transceiver for vessel pulsation monitoring, but it is not capable of Doppler imaging (Supplementary Discussion 25)^70^. Developing a more advanced circuit with multiple transceivers for Doppler imaging would enhance patient mobility, help assess the impact of maternal posture and physical activity on fetal health, and show how maternal circulation affects fetal hemodynamics^71^.

Third, further investigations are necessary to establish the UPatch as a diagnostic tool to identify specific conditions or diseases^72^. Continuous hemodynamic monitoring with a particular focus on the frequency and duration of critical changes (such as decreased or absent end diastolic velocity), is essential for assessing the severity of hypoxia and identifying optimal intervention windows to prevent adverse perinatal outcomes^73^. The UPatch can define individualized baselines that characterize the normal range of short-term fluctuations for each patient while highlighting patterns of sustained abnormalities that indicate evolving pathology. This personalized calibration reduces false alarms triggered by benign transient events and allows clinicians to focus on persistent deviations that truly indicate risk. These studies could establish the real-world clinical utility of the UPatch.

Finally, this study was limited to using only the UPatch. Future work could focus on integrating UPatch data streams (such as fetal heart rate and Doppler indices) with additional physiological metrics from both the fetus (such as electrocardiogram) and the mother (such as blood pressure, pulse oximetry, and tocodynamometry). Such multimodal integration would provide a more comprehensive and accurate understanding of the dynamic interactions between fetus and mother^15^. Additionally, applying big data analysis to correlate UPatch data streams with postnatal and postmortem results would yield unprecedented insights into the mechanisms of fetal complications, advancing diagnosis and intervention strategies^74^.

## Methods

### Fabrication of the UPatch

The fabrication process can be divided into three steps: (1) transducer assembly, (2) electrode printing and acoustic lens integration, and (3) device packaging.

#### (1) Transducer assembly:

A silicone (Ecoflex–0030, Smooth–On) layer was first spin-coated at 3000 rpm for 60 s on two glass slides and cured in an 80 °C oven as temporary substrates. Conductive epoxy (Von Roll 3022 E–Solder, EIS) was prepared as the backing layer by mixing an Ag–epoxy composite with a hardener in a 12.5:1 weight ratio and subsequently degassed in a vacuum desiccator. Then, a 1–3 composite (Del Piezo Specialties) was placed on the silicone surface of one glass slide. The degassed conductive epoxy was placed on top and covered by the other silicone-coated glass slide, followed by curing at room temperature for 24 h. The thickness of the backing layer (400 μm in this study) was controlled by placing certain spacers in between the two glass slides.

After curing, the glass slides were removed, and the transducer made of 1–3 composite/backing was diced. To ensure direct transfer of the backing layer side of the transducer to the signal electrodes, the 1–3 composite side of the transducer was first placed on an ultraviolet tape (UHP–110M3, Denka) with a frame film applicator (UH114, Ultron Systems, Inc.). Then, the transducer was diced with a dicing machine (DAD3221, Disco) to 25.55 mm by 12 mm using a diamond blade (ZH14–SD1700–V1–90 GE, Disco) at 25,000 spindle rotations per minute (rpm) and 0.5 mm/s feed speed. A second diamond blade (Z09–SD4000–Y1–60, Disco) was used to dice the 0.05 mm kerf of the transducer. Super multipass dicing was performed with 50 μm depth stepwise cuts at 50,000 spindle rpm and 10 mm/s feed speed to minimize blade and transducer vibration during dicing (Supplementary Fig. 2 and Supplementary Discussion 7). Finally, the diced transducers were detached from the ultraviolet tape by an ultraviolet curing system (UH104, Ultron Systems Inc.).

#### (2) Electrode printing and acoustic lens integration:

The substrate for electrode printing was prepared by spin-coating polydimethylsiloxane (Sylgard 184) at 3000 rpm for 60 s on a glass slide, followed by curing in an 80 °C oven as a temporary substrate. Then, polyimide/copper laminates (AC181200EN, DuPont) and the polydimethylsiloxane–coated glass slide were activated by ultraviolet light (PSD series Digital UV Ozone System, Novascan) and bonded together. Electrode patterns (such as electromagnetic shielding, mesh ground electrode, and two signal electrodes) designed with AutoCAD (Autodesk) were laser ablated (G4 Pulsed Fiber Laser, wavelength 1095 to 1065 nm, energy 0.228 mJ, frequency 35 kHz, speed 300 mm/s, and pulse width 500 ns) on the polyimide/copper laminate (Supplementary Fig. 9). Two polyimide masks were prepared by laser ablation for dispensing conductive epoxy on the transducer bonding pads of the two signal electrode layers.

For acoustic lens integration, an aluminum block mold was designed with Solidworks (Dassault Systemes) and machined using a computer-numerically controlled machining (Tormach 1100MX, Tormach Inc.) at a spindle speed of 10,000 rpm and feed rate of 40 in/min (Supplementary Fig. 6 and Supplementary Discussion 8). The mold was cleaned with isopropanol before silicon elastomer was cured in the mold and used as the acoustic lens.

#### (3) Device packaging:

First, polyimide tapes were adhered to two glass slides to ensure device detachment after final packaging. Silicone (Ecoflex–0030, Smooth–On) mixed with black dye was spin–coated on the polyimide tape at 4000 rpm for 60 s, followed by curing at room temperature for 2 h. The electromagnetic shielding layer, which was transferred onto a water–soluble tape (AQUASOL), and the silicone–coated glass slide was activated by ultraviolet light and bonded together, followed by heating in an 80 °C oven for 30 min. After the water–soluble tape was dissolved, a silicone layer was spin–coated on top at 4000 rpm for 60 s followed by curing. Similarly, the first layer of the signal electrode was aligned and transferred onto the electromagnetic shielding layer via a water–soluble tape. A polyimide mask was placed on the signal electrode to protect the transducer bonding pads of the electrode from another silicone layer spin–coated on top. After the polyimide mask was removed and the silicone layer was cured, the second layer of the signal electrode was aligned and stacked onto the first layer of the signal electrode. Conductive epoxy was dispensed onto the transducer bonding pads of both signal electrodes through a polyimide mask. After the mask was removed, the backing layer side of the diced transducers was aligned and bonded by curing the conductive epoxy for 8 h at room temperature and then 2 h at 40 °C. Flexible printed circuit cables were bonded to the signal electrodes via solder paste (Sn_42_Bi_57.6_Ag_0.4_, m.p. 138 °C).

The mesh ground electrode was bonded to a silicone–coated glass slide. After conductive epoxy was dispensed using a polyimide mask, the mesh ground electrode was aligned and cured onto the transducers. The exterior boundaries of the mesh ground electrode and electromagnetic shielding layer were connected with vertical interconnect accesses by soldering copper wires (Remington Industries) to create a Faraday cage (Supplementary Fig. 10 and Supplementary Discussion 9)^75^. After the device was fully encapsulated with silicone elastomer, the glass slides were removed. Finally, the acoustic lens was aligned and bonded with silicone elastomer onto the packaged transducers.

### Quality assurance

The UPatch was transmitted at a frequency of 2.5 MHz with a sampling rate of 10 MHz. Apodization was uniform across 64 channels with the imaging view angle of 45°. The number of compounding angles was 21 with a step size of 0.75° for B-mode imaging. The ensemble length was 14 for color flow mode. Doppler modes utilized a pulse repetition frequency of 3000 Hz. The sample gate size for spectral Doppler was 2 mm.

The UPatch was characterized using two phantom setups and compared to a handheld clinical ultrasound device (P4–1, ATL), both transmitting at 2.5 MHz. Both the UPatch and the handheld clinical ultrasound device (P4–1, ATL) were connected to the Vantage 256 (Verasonics), rather than connecting the P4–1 device to a clinical backend system (Voluson E10, GE). This method allows comparison of the probe performance more fairly and avoids confounding effects from differences in backend signal processing.

First, the brightness mode was characterized with a multipurpose phantom (CIRS ATS 539, CIRS Inc.) (Supplementary Fig. 15)^40,41^. Monofilament wires were used to determine the axial and lateral resolutions. A volume of water on top of the phantom was used as a standoff to characterize depths up to 20 cm. The distance between two adjacent pixels in the axial and lateral directions was calculated as:

(1) $$\Delta y=\frac{depth}{N_{pixel,axial}\;-\;1}$$

(2) $$\Delta x=\frac{depth}{N_{pixel,lateral}\;-\;1}$$

The full width at half maximum of the point spread function for each wire in the axial and lateral directions was measured. The axial and lateral resolutions were calculated by multiplying the number of pixels within the full width at half maximum and adjacent pixel distance. The six tissue–mimicking contrast targets in the phantom were used to characterize the dynamic range and contrast-to-noise ratio^41^. The dynamic range was calculated by linearly fitting among the six greyscale targets and finding the maximum positive and negative contrasts corresponding to grey values of 255 and 0. Then, the negative one was subtracted from the other. The contrast-to-noise ratio was calculated by:

(3) $$\mathrm{Contrast}-\mathrm{to}-\mathrm{noise}\;\mathrm{ratio}=\frac{\left|\mu_{\mathrm{in}}\;-\;\mu_{\mathrm{out}}\right|}{\sqrt{\sigma_{\mathrm{in}}^{2}\;+\;\sigma_{\mathrm{out}}^{2}}}$$

where $\mu_{in}$ and $\sigma_{in}$ are the mean and standard deviation of pixel intensities within each target, and $\mu_{\mathrm{out}}$ and $\sigma_{\mathrm{out}}$ are the mean and standard deviation of pixel intensities outside each target. The contrast and contrast-to-noise ratio were computed based on displayed images rather than raw radiofrequency signals or in-phase and quadrature (I/Q) data, meaning that the values were derived from log-compressed images ^76^.

The color and spectral Doppler modes were characterized with Doppler fluid (769DF, CIRS Inc.) pumped into a Doppler phantom (CIRS ATS 523A, CIRS Inc.) with 2, 4, 6, and 8 mm diameter using a peristaltic pump (Supplementary Fig. 16)^42^. The UPatch was positioned at varying distances from 30 mm to 120 mm at intervals of 10 mm from the phantom. The signal-to-noise ratio in the color flow images was calculated by increasing the color gain to maximum to obtain a noise level in the tissue mimicking phantom. The accuracy of spectral Doppler was measured by comparison to a known velocity from the peristaltic pump at different depths and velocities.

### Acoustic exposure and thermal measurement protocols

#### (1) Hydrophone:

A needle hydrophone (HNP–0400, ONDA) was connected to a right-angle adapter (ONDA), pre-amplifier (AH–2010–100, ONDA), and DC block (BNP, ONDA) and suspended in deionized water (Supplementary Fig. 24)^77^. The signals were read with an oscilloscope (PicoScope 5000 Series, Pico Technology, Cambridgeshire). At 2.5 MHz, the effective sensitive element diameter (a more meaningful measure of spatial resolution than the geometrical sensitive element diameter, which was 400 μm) was estimated to be 480 μm^78^. The uncertainties of hydrophone-based pressure, intensity, and power measurements are ~ 15%^79^.

The hydrophone system was calibrated < 1 year before the measurement. The calibration $M_{c}(f)$ is the hydrophone end–of–cable open circuit sensitivity, which can be converted by:

(4) $$M_{C}{\left(f\right)}_{V/Pa}=10^{\frac{M_{C}{\left(f\right)}_{dB\;re.1V/\mu Pa}+120}{20}}$$

where $M_{C}{\left(f\right)}_{V/Pa}$ is the sensitivity in volts per pascal and $M_{C}{\left(f\right)}_{dB\;re.1V/\mu Pa}$ is the sensitivity in decibels relative to 1 volt per micropascals^77^. The manufacturer’s sensitivity magnitude data (i.e., relative calibration from 1 to 20 MHz) was used to perform magnitude–based waveform deconvolution to compensate for the hydrophone’s nonuniform frequency response in the signal spectrum (Supplementary Fig. 24). Magnitude–based deconvolution has been shown to be nearly as effective as complex deconvolution for improving the accuracy of pressure and intensity measurements^80^. Additionally, because the ratio of the hydrophone sensitive element diameter $d_{g}$ to the product of the wavelength λ and F/# (ratio of focal distance to aperture width) << 1, a hydrophone spatial averaging correction was not necessary^81^.

Considering the capacitive load of the preamplifier when converting the voltage measurements to pressure values, the loaded sensitivity $M_{L}(f)$ can be estimated as:

(5) $$M_{L}(f)=G(f)M_{C}{\left(f\right)}_{V/Pa}\frac{C_{H}}{C_{H}\;+\;C_{A}\;+\;C_{c}}$$

where G(f) is the preamplifier gain (20 dB in this study), $C_{H}$ is the capacitance of hydrophone (70 pF in this study), $C_{A}$ is the capacitance of preamplifier (7 pF in this study), and $C_{C}$ is the capacitance of right–angle adapter (1.6 pF in this study)^77^.

A hydrophone controlled by a 3D linear motor in a water tank was used to measure the acoustic signal intensity (Supplementary Fig. 24). Transverse scans with scanning increments of Δx = 0.5 mm (approximately one effective hydrophone diameter) and Δy = 0.5 mm and an axial scan with scanning increments of Δz = 0.5 mm from break–point depth to focal depth were performed (Supplementary Discussion 11)^82^. Two different beamforming methods were measured separately: diverging beam (for brightness and color flow mode) and focused beam (for spectral Doppler mode) that was focused at every 1 cm depth (between 5 to 15 cm) along the beam axis. The UPatch was activated at 30 V with a pulse repetition frequency of 3000 Hz. Power was derived from the hydrophone by integrating over a transverse plane within the beam cross–sectional area where the intensity was at least −26.2 dB of the peak value. All measurements were calculated based on the settings used for duplex imaging and spectral Doppler because these were the modes utilized during continuous monitoring in this study.

#### (2) Radiation force balance:

Power measurements were obtained from a radiation force balance (RFB–2000, ONDA, Sunnyvale, CA) with a flat absorbing target, which could accurately measure power levels up to 2 W (Supplementary Fig. 25)^83^. First, a 1 g weight was used to calibrate the radiation force balance. The calibration constant obtained was 0.9699 A/N, which fell in the range designed by ONDA, 0.9–1.1 A/N. The distance between the radiation force balance and the UPatch was set to ≥ 1 cm to avoid thermal and electrical coupling effects between the radiation force balance and the UPatch. Acoustic output power was obtained from the entire aperture of the UPatch, and the bounded–square output power over a 1 cm^2^ area was measured by masking the UPatch.

The thermal index was calculated based on acoustic output power measurements from both the radiation force balance and hydrophone scan in the transverse plane, providing two independent measurements to minimize uncertainty in the calculation^49^.

#### (3) Surface temperature testing:

The surface temperature of the UPatch was evaluated under two scenarios to simulate clinical conditions based on IEC 60601–2-37 (ref. ^84^). Although the standard requires only 30-min testing^84^, we extended the assessments to 48 h to ensure compliance with regulatory standards even under prolonged operation.

First, a phantom test was performed to replicate the setup in the hospital (Supplementary Fig. 23). The phantom consisted of a silicone-based skin (NPL, UK) and agar-based tissue (NPL, UK) with acoustic and thermal properties similar to those of human skin and tissue, respectively. A type K thermocouple (5SRTC-TT-KI-40–1M, OMEGA, Norwalk, Connecticut) with a thickness of 75 μm (<< ultrasound wavelength at 2.5 MHz) was placed on top of the skin mimicking phantom to measure the surface temperature of the UPatch^48^.

Second, to evaluate thermal changes when the UPatch was activated in air, surface temperature was measured. Because the UPatch/air interface has a higher acoustic impedance mismatch compared to that of the UPatch/tissue phantom interface, more heat is expected to be generated in the air^48^. The UPatch was suspended on a clean surface in a stationary position with minimal airflow across the transducer area. The same thermocouple was adhered to the UPatch for temperature measurement.

### Evaluation of the vessel–tracking algorithm

The comparative analysis of the lateral and axial discrepancies in defining the sample gates between the tracking algorithm and a sonographer was performed using double–blind method (Supplementary Discussion 18). The tracking algorithm and sonographer independently selected sample gates from 1,000 duplex images. The lateral and axial discrepancies were calculated between the two sample gates (Fig. 3d). A 2 mm sample gate length is commonly employed in hospitals (Supplementary Discussion 19)^6^. Thus, within a 2 mm discrepancy between the tracking algorithm and the sonographer, the sample gate was considered to acquire spectral Doppler signals accurately.

Additionally, a new dataset was acquired, containing 500 duplex images from scanning the clinical device on a participant and 500 duplex images from the UPatch on another participant. The tracking algorithm identified the sample gates in all 1,000 images. We engaged three experienced sonographers, each with 30, 8, and 20 years of fetal ultrasonography experience, to independently assess the tracking performance on 1,000 images (Supplementary Fig. 39). This validation method aligns with the recently FDA-cleared cardiac Doppler tracking algorithm^85^, which also relied on three sonographers and evaluated only 168 images. All three sonographers evaluated the tracking algorithm with a customized graphical user interface in Matlab R2023b (Mathworks). “Optimal” was defined by the sonographer as the tracked sample gate that could potentially be used for spectral Doppler acquisition, “Sub-optimal” was defined as the sample gate potentially providing low-quality spectral Doppler signals, and “Non-diagnostic” was defined as the sample gate that could not provide any spectral Doppler signal (Supplementary Fig. 39 and Supplementary Discussion 18).

Finally, the tracking algorithm was evaluated with the UPatch based on the systolic-to-diastolic ratio of a participant (Supplementary Discussion 18). Each pair of measurements was acquired sequentially by a sonographer and the tracking algorithm.

### Human test protocol

All human tests were approved by the Institutional Review Board of the University of California San Diego (#804817) and the UK Research Ethics Committee (23/WA/0032). The participants all gave voluntary consent to the UPatch tests, and all measurements were conducted by a clinician. The study included 62 participants for accuracy validation (Fig. 2f, Table 1, and Supplementary Fig. 32) and 52 participants for continuous monitoring (Supplementary Figs 40–95). This dataset captures a broad range of clinical conditions across the study cohort, including variations in fetal biometry (e.g., small and large for gestational age) (Fig. 4e–i and Supplementary Figs 96 and 97) and gestational age (Table 1). For the validation test, three pairs of measurements were acquired from the UPatch and a handheld clinical ultrasound device (Voluson E10, GE) on 62 participants. For continuous monitoring, the clinical device was used to locate the position of the vessel. Then, the UPatch was taped on the maternal abdomen for continuous monitoring. The current study was conducted under inpatient conditions, where pregnant participants were free to move within the limits of standard clinical monitoring. During the recordings, the pregnant participants naturally moved within their beds, accompanied by various fetal behavioral states, fetal movements, and uterine contractions.

The UPatch was designed for easy repositioning as needed. It is movable, can be sanitized with alcohol wipes, and can be repositioned without compromising functionality. This allows adaptability in clinical use while maintaining hygiene and patient comfort.

For fetal biometry, estimated fetal weight (EFW) was calculated based on Hadlock’s IV formula^51^:

(6) EFW=1.3596-0.00386AC·FL+0.0064HC+0.00061BPD·AC+0.0424AC+0.174FL

where AC is abdominal circumference, FL is femur length, HC is head circumference, and BPD is biparietal diameter.

## Supplementary Material

Supplementary Files

This is a list of supplementary files associated with this preprint. Click to download.

• ParkSupplementaryInformation.pdf

• ParkSupplementaryVideo1.mp4

## Figures and Tables

**Table 1 |:** Participant demographics in the validation study. The demographic information includes race/ethnicity, age, height, weight, body mass index, gestational age, and placental location at the time of the study. The validation was performed on a diverse participant cohort, ensuring the broad applicability of the UPatch.

Race/ethnicity	n (percentage)

White	45 (72.6%)
Asian	5 (8.1%)
Mixed, American Indian	8 (12.9%)
Unknown*	4 (6.4%)

At the time of study	Mean ± standard deviation

Age (years)	31.45 ± 4.88
Height (cm)	164.44 ± 7.34
Weight (kg)	73.33 ± 18.11
Body mass index	27.17 ± 6.92
Gestational age (days)	238.90 ± 26.68

Placental location	n (percentage)

Anterior	36 (58.1%)
Posterior	17 (27.4%)
Lateral	4 (6.5%)
Fundal	5 (8.1%)

* The participants refused to provide this information.

## Data Availability

The data supporting the findings of this study are available in the main text or the Supplementary Information.
